# mTORC1 signalling and eIF4E/4E-BP1 translation initiation factor stoichiometry influence recombinant protein productivity from GS-CHOK1 cells

**DOI:** 10.1042/BCJ20160845

**Published:** 2016-12-09

**Authors:** Lyne Jossé, Jianling Xie, Christopher G. Proud, C. Mark Smales

**Affiliations:** 1 Centre for Industrial Biotechnology and School of Biosciences, University of Kent, Canterbury, Kent CT2 7NJ, U.K.; 2 South Australian Health and Medical Research Institute, PO Box 11060, SA5001 Adelaide, Australia; 3 School of Biological Sciences, University of Adelaide, Adelaide, SA, Australia

**Keywords:** bioprocessing, Chinese hamster ovary cells, mechanistic target of rapamycin, monoclonal antibodies, mRNA translation, recombinant proteins

## Abstract

Many protein-based biotherapeutics are produced in cultured Chinese hamster ovary (CHO) cell lines. Recent reports have demonstrated that translation of recombinant mRNAs and global control of the translation machinery via mammalian target of rapamycin (mTOR) signalling are important determinants of the amount and quality of recombinant protein such cells can produce. mTOR complex 1 (mTORC1) is a master regulator of cell growth/division, ribosome biogenesis and protein synthesis, but the relationship between mTORC1 signalling, cell growth and proliferation and recombinant protein yields from mammalian cells, and whether this master regulating signalling pathway can be manipulated to enhance cell biomass and recombinant protein production (rPP) are not well explored. We have investigated mTORC1 signalling and activity throughout batch culture of a panel of sister recombinant glutamine synthetase-CHO cell lines expressing different amounts of a model monoclonal IgG4, to evaluate the links between mTORC1 signalling and cell proliferation, autophagy, recombinant protein expression, global protein synthesis and mRNA translation initiation. We find that the expression of the mTORC1 substrate 4E-binding protein 1 (4E-BP1) fluctuates throughout the course of cell culture and, as expected, that the 4E-BP1 phosphorylation profiles change across the culture. Importantly, we find that the eIF4E/4E-BP1 stoichiometry positively correlates with cell productivity. Furthermore, eIF4E amounts appear to be co-regulated with 4E-BP1 amounts. This may reflect a sensing of either change at the mRNA level as opposed to the protein level or the fact that the phosphorylation status, as well as the amount of 4E-BP1 present, is important in the co-regulation of eIF4E and 4E-BP1.

## Introduction

Cultured mammalian cell expression systems, particularly Chinese hamster ovary (CHO) cells, are used to manufacture high-value biotherapeutic recombinant proteins (rPs) such as monoclonal antibodies (mAbs) [1]. Although the ability of such cell manufacturing ‘factories’ to produce rP products has been advanced over the last two to three decades, there is still an interest in further optimising the yields that can be delivered for novel and difficult to express proteins and the quality/homogeneity of the protein product. Two key parameters determining the amount of recombinant protein an expression system generates are (i) the maximum viable cell concentration achieved and the length of time this is maintained (the integral of viable cell concentration or IVC) and (ii) the cell-specific productivity of the cell (qP, or the amount of recombinant protein a cell manufactures per unit time, usually expressed as pg/cell/day). In this regard, improved growth medium and feeding strategies have resulted in dramatic increases in the maximum viable cell concentration and IVC achievable for mammalian cells in the bioreactor, and this has been associated with extended culture viability and enhanced recombinant protein yields [2]. Indeed, achieving high yields of recombinant protein of a clinically acceptable quality is dependent on a multiplicity of parameters, including the choice of the host expression system, the achieved viable cell mass [3,4], gene copy number [5], site-specific integration [6,7], the cellular processes responsible from gene to protein in the synthesis of the desired product [1,8], the bioreactor environment (e.g. nutrients and oxygen levels) [2,9], the authenticity and homogeneity of the product [10,11] and the yield and success of downstream processing [12,13].

There are now many reports suggesting that major cellular constraints upon recombinant protein production (rPP) can be post-transcriptional ([14–16]). One such control point is mRNA translation, with many reports having now reported that global and specific mRNA translation is a key parameter influencing rPP yields [14–16]. Other reports have suggested that ribosome biosynthesis also influences recombinant protein yields [17], and these reports collectively demonstrate that control of mRNA translation and ribosome biogenesis are important factors in cell growth and recombinant protein yield from mammalian cells. mRNA translation (protein synthesis) is catalysed by ribosomes, and mammalian target of rapamycin complex 1 (mTORC1) is a master regulator of ribosome biogenesis as well as mRNA translation [18]. With respect to the growth of recombinant cell lines, mTORC1 regulates these processes via the co-ordination of signalling pathways in response to growth factors, nutrient availability (amino acids), intracellular energy status (ATP levels) and diverse cell stresses [18], all factors that play key roles in regulating recombinant protein yields from mammalian cells. mTORC1 is thus likely to be a key global regulator of exactly those properties that are essential to achieving and maintaining high-level rPP from mammalian cells.

mTOR is the catalytic subunit of two functionally distinct complexes, mTORC1 and mTORC2. mTORC1 senses extra- and intra-cellular signals whereby growth factors, nutrients and energy promote mTORC1-dependent cell growth/proliferation and protein synthesis. At the same time, mTORC1 promotes ribosome biogenesis by enhanced transcription of ribosomal RNAs and translation of mRNAs for ribosomal (r-)proteins to increase protein synthetic capacity. Reduced mTORC1 activity leads to activated macroautophagy, which mediates the breakdown of cellular components into building blocks (e.g. amino acids and other small molecules) that may compensate for deficient nutrient supply [18]. The mTORC1 component raptor binds ribosomal S6 protein kinase 1 (S6K1) and eukaryotic initiation factor 4E-binding protein 1 (4E-BP1), thereby recruiting these substrates to be phosphorylated by mTOR. When hypophosphorylated, 4E-BP1 binds to eIF4E and prevents it from interacting with eIF4G to promote ribosome recruitment to mRNAs. It can thus repress the initiation of mRNA translation. By directly phosphorylating 4E-BP1 at multiple sites (in human 4E-BP1, Thr^37/46^, Thr^70^, Ser^65^), mTORC1 promotes its dissociation from eIF4E allowing the formation of the eIF4F complex and the initiation of cap-dependent translation [18]. Recent work has shown that increased 4E-BP1 phosphorylation is correlated with enhanced interferon-γ production in CHO cells. The authors suggest that this was due to the alleviation by mTORC1 of the repression of translation initiation [19].

With respect to rPP in mammalian cells, exogenous expression of mTOR has been reported to simultaneously improve key processes underpinning rPP from CHO cells, including cell growth, proliferation, viability and cell-specific productivity [20]. A further study has reported that in plasma cells (the cells that ‘naturally’ synthesise and secrete Igs), protein synthesis is regulated by cross-talk between endoplasmic reticulum stress and mTORC1 signalling [21]. Others [22] report that rapamycin differentially targets S6K and 4E-BP1, two downstream effectors of mTORC1. Here, we have examined the expression and phosphorylation state of many key proteins involved in mTORC1 signalling, as well as downstream effectors, using western blot analysis of CHO cell lysates collected throughout culture of cell lines with different productivity characteristics. Our data show that the flux of protein synthesis was altered across the culture time course reflecting mTORC1 signalling in the different cell lines. 4E-BP1 protein amounts were found to be elevated in a low-producer cell line alongside eIF4E amounts. We therefore sought to explore whether the relative amounts of eIF4E and 4E-BP1 influence the phenotype of cell lines (their productivity) in an initial set of four cell lines and a larger pool of cell lines [15]. These data show that the eIF4E/4E-BP1 translation initiation factor stoichiometry relates to recombinant protein productivity from glutamine synthetase (GS)-CHOK1 cells, and we discuss the implications of this for cell line engineering approaches.

## Materials and methods

### Materials

Materials were obtained from Sigma–Aldrich unless otherwise indicated below.

### Methods

#### Cell culture and general sample preparation

GS-CHOK1 cells were from Lonza Biologics. Cells were grown in CD-CHO medium (Invitrogen) supplemented with 25 µM l-methionine sulfoximine. Cells were passaged three times prior to seeding 100 ml cultures for each cell line in 500 ml Erlenmeyer shaking flasks at 0.3 × 10^6^ viable cells/ml. Cell counts were performed daily using a Vi-CELL 1.01 instrument (Beckman Coulter) to determine total and viable cell concentrations using the trypan blue dye exclusion method. Samples were taken each day (for 8–11 days), until cultures dropped below 60% viability. At each sampling point, 1 × 10^7^ viable cells were removed, centrifuged at 1000 rpm for 3 min at 4°C and the supernatant removed (and immediately frozen at −20°C). The pellet was lysed in 200 µl of western lysis buffer [20 mM Tris–Cl (pH 7.5), 10 mM EDTA, 10 mM EGTA, 150 mM NaCl and 1% (w/v) Triton and 2 µl of protease/phosphatase inhibitor cocktail 100× (New England Biolabs)]. Samples were further centrifuged at 13 000 × ***g*** for 2 min at 4°C in order to sediment cell debris. The cytosolic fractions were then transferred to a fresh tube and sample buffer was added. The protein extracts were immediately stored at −20°C.

#### ^35^S-methionine incorporation assay

Viable cells (2 × 10^6^) in 2 ml of medium were labelled with 762 kBq of [^35^S]methionine (PerkinElmer) in CD-CHO medium (Invitrogen) for 1 h, washed once with PBS and lysed in buffer containing 1% Triton X-100, 1 mM EDTA, 50 mM Tris–Cl, 1 mM EDTA, 0.1% β-mercaptoethanol, 1× protease/phosphatase inhibitor cocktail (#5872, Cell Signaling Technology).

#### Pull-down assay using γ-aminophenyl-7-methyl-guanosine 5′-triphosphate agarose

Immobilised γ-aminophenyl-7-methyl-guanosine 5′-triphosphate (m^7^GTP)-agarose was purchased from Jena Bioscience. Beads (#AC-155S) were incubated with fresh CHO cell extracts in buffer containing 1% Triton X-100, 1 mM EDTA, 50 mM Tris–Cl, 1 mM EDTA, 0.1% (v/v) β-mercaptoethanol, 1× protease/phosphatase inhibitor cocktail (# 5872, Cell Signaling Technology) at 4°C for 2 h and then washed three times with cold PBS buffer. The proteins attached to the washed agarose were then subjected to 16% SDS–PAGE followed by western blotting.

#### Gene silencing by siRNA

Custom-made Stealth siRNAs were purchased from Invitrogen. Cells were seeded in six-well plates at a density of 750 000 cells/well and transfected with 4.5 (CHO-42) or 6.0 µl from a 20 nM siRNA pool against Chinese Hamster 4E-BP1 using Lipofectamine LTX (Invitrogen). Cell extracts were examined 48 h after transfection. For protein phosphatase magnesium-dependent 1 gamma (PPM1G), gene silencing was carried out using a 20 nM RNA Max stock from Eurofins and cells were transfected with Hi-Perfect (Qiagen).

#### SDS–PAGE and western blot analysis

Proteins were run on Tris–glycine gels [6, 10 and 16% (w/v) acrylamide, depending on the protein of interest]. After transfer to the polyvinylidene difluoride membrane, bound antibodies were detected using standard Enhanced Chemiluminescence analysis. Anti-β-actin antibodies (all diluted at 1/5000) were purchased from Sigma–Aldrich. Anti-4E-BP1 (clone 5H11) and eIF4G antibodies were purchased from Cell Signaling Technology. Secondary antibodies were either horseradish peroxidase-conjugated anti-rabbit or anti-mouse (both from Sigma–Aldrich). Anti-eIF4E antibodies were a kind gift from Prof. Simon Morley (Sussex). Phospho-S6 ribosomal protein (Ser240/244) (D68F8) XP rabbit mAb was purchased from Cell Signaling Technology.

#### Immunofluorescence microscopy

Prior to the addition of CHO42 and CHO52, sterile circular coverslips were deposited into 24-well plates and coated with Corning Cell Tak Adhesive (at a concentration of 35 µg per ml, making sure the pH was in the range of 6.5–8). A 150 µl aliquot of a mid-exponential culture was added to the well. Following attachment, the cells were immediately fixed with 4% paraformaldehyde and permeabilised with 0.5% Triton in 1× PBS. All primary and secondary antibodies used in the present study were diluted 1/100 in 1% goat serum in 1× PBS. Goat anti-rabbit IgG (whole molecule)–TRITC (tetramethyl rhodamine isothiocyanate) antibody and goat anti-mouse were purchased from Sigma–Aldrich. Coverslips were mounted on slides with Vectashield with or without DAPI (at a final concentration of 0.1 µg/ml).

## Results

### Characterisation of growth and mAb production profiles in model GS-CHOK1SV antibody producing cell lines

**Figure 1.**
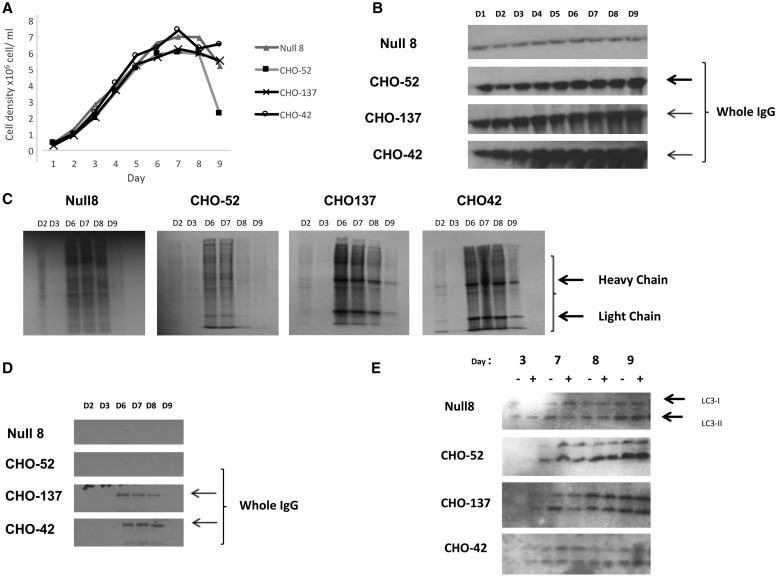
Comparison of cell productivity and translation rate in parental (Null 8) and mAbs producing cell lines (CHO42, CHO52 and CHO137). (**A**) For all cell lines, the growth profile was measured over the course of 9 days. (**B**) mAb secretion was also analysed in parallel by western blot analysis of the culture supernatant. (**C**) The overall rate of mRNA translation between cell lines was compared by adding 760 kBq of l-[^35^S] methionine to 2 × 10^6^ live cells for 1 h, followed by a PBS wash. The neosynthetised proteins were separated using standard SDS–PAGE and were revealed on X-ray film autoradiography. The bands assigned as heavy and light chains are indicated by arrows. (**D**) The rate of appearance of mAbs was also examined following the procedure described in **C**, except that the cell fraction was incubated with protein A beads prior to gel separation. (**E**) Autophagy. The monitoring of the autophagosomal marker LC3-II was performed in the presence or absence of the lysosomal inhibitor chloroquine.

Clonally derived recombinant GS-CHOK1 cell lines expressing a model mAb [22,23] were grown over the course of 9 days under batch culture conditions. The cell lines were selected for, and exhibited, different growth (Figure 1A) and productivity characteristics. For example, the viable cell number in the CHO52 cell line declined from day 8 to day 9 much more than the other cell lines. In terms of productivity, Null8 is a non-producing cell line that has been through the same GS selection process as the mAb-producing cell lines, but lacks the heavy and light chain IgG genes, while CHO52 was ranked as a low producer and CHO137 and CHO42 were considered high producers for the present study with their estimated specific production rates (pg/cell/h), having previously been estimated as 0.032, 0.49 and 0.31, respectively [8]. Western blot analysis for the amount of mAb in the cell culture supernatant on different days throughout culture confirmed the relative productivities of these cell lines, with CHO42 being the highest producer and CHO52 the lowest (Figure 1B). Null8 exhibits a background, non-specific binding when analysed by western blot (Figure 1B), which remains unchanged across the time course. The overall global protein synthesis rates at various time points throughout batch culture were compared by measuring the incorporation of [^35^S] methionine into newly synthesised intracellular proteins. Our results show that the peak of translational activity occurred during the middle of the batch culture (Figure 1C), that is, towards the end of the growth phase and into the stationary phase of the culture (days 6–8, Figure 1C). The majority of mAb was produced and assembled across the same period, as shown by a protein A pull-down experiment of the intracellular [^35^S]-labelled mAb carried out on the same total extract in Figure 1C (see Figure 1D).

### Autophagy is activated towards the end of batch culture in GS-CHOK1 cells

In the same cell line panel, we also investigated the intracellular accumulation of autophagy markers. Autophagy can reflect nutrient deprivation and activation of autophagy is a known strategy to preserve cellular fitness [24]. LC3-II amounts were therefore determined in cell lysates from the different cell lines throughout culture in the presence and absence of chloroquine by western blotting. These analyses showed that, in the CHO52 cell line, the least productive line and the one where cell viability decreased earlier than the other cell lines investigated, the onset of autophagy was observed earlier than in the Null8 and high-producing cell lines, as indicated by the increased amount of the modified LC3 (derived by lipidation of LC3-I to generate LC3-II; Figure 1E). This suggests that autophagy as a result of cellular stress, possibly nutrient deprivation, is activated earlier in the CHO52 cell line than the other cell lines investigated.

### Translation initiation factors undergo an iterative series of phosphorylation changes throughout batch culture

**Figure 2.**
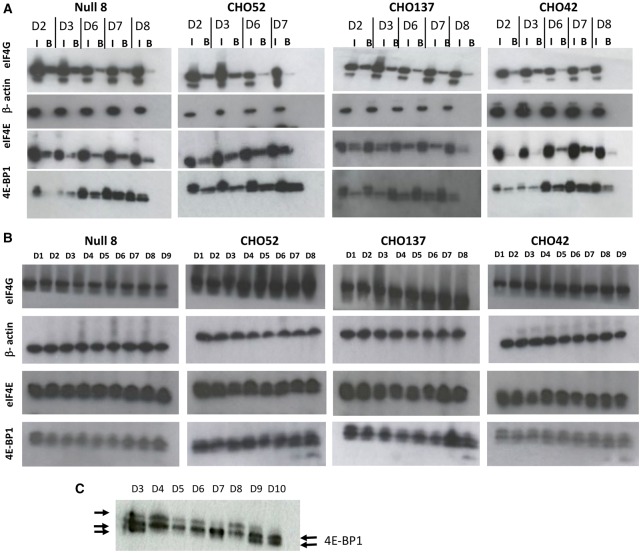
Qualitative and quantitative analysis of factors regulating the translation initiation in parental and mAb-producing cell lines. (**A**) Purification of mRNA cap-binding proteins using m^7^GTP-agarose beads using total cell extracts, done on different days (D) as stated. For each day, the panel shows SDS–PAGE/western blot analysis of the 4E-BP1, eIF4E, eIF4G and β-actin proteins in the input (I) and bound (B) fractions. (**B**) Total proteins were separated by SDS–PAGE, and the levels of total 4E-BP1, eIF4E, eIF4G and β-actin were examined by western blot analysis on stated days (D). (**C**) To detect faster running bands, D3 and D4 from CHO137 were obtained from an independent time course and separated at greater resolution.

4E-BP1 competes with the scaffold protein eIF4G for binding to the translation initiation factor eIF4E, and this is modulated by the phosphorylation of 4E-BP1, which decreases its affinity for eIF4E [25]. Therefore, the interaction of eIF4E and eIF4G is related to the translation activity (on or off). eIF4E and the proteins to which it binds, 4E-BP1 or eIF4G, can readily be purified from cell lysates by affinity chromatography using the 5′-cap analogue m^7^GTP immobilised on agarose beads. The input of total cell extracts and the proteins isolated using m^7^GTP-agarose beads were analysed by western blotting (Figure 2A). This revealed that differently phosphorylated forms of 4E-BP1 could be detected particularly from day 6 of the time course. The signal from the faster migrating form became stronger towards the end of the time course. In Figure 2C, a higher resolution gel shows that 4E-BP1 displays slow and fast migrating species, which correspond to different phosphorylated forms of 4E-BP1. 4E-BP1 undergoes phosphorylation transitions between these forms, with the lower migrating forms corresponding to a slower translational rate (early and late in the time course). These results mirror those obtained from the [^35^S]methionine labelling experiment and are in accordance with other studies [1] that indicate that a change in the phosphorylation of 4E-BP1 (hyperphosphorylation) relates to protein synthesis rates. The amount of eIF4G bound to the m^7^GTP-agarose beads decreased sharply on day 8 of the batch cultures (or day 7 for the low producer, Figure 2A), suggesting that less eIF4G can be bound to eIF4E to mediate translation initiation. The western blots for eIF4E for the line CHO137 suggested the presence of two bands, although these were not well resolved in the other samples where a broader single band was observed (Figure 2A).

We also investigated the amounts of total 4E-BP1, eIF4E and eIF4G proteins at each point of the time course (Figure 2B,C). Their total levels did not change appreciably across the time course (Figure 2B). However, the low-producer CHO52 cell line appeared to have higher amounts of both 4E-BP1 and eIF4E compared with the other three cell lines when compared with the β-actin loading control (Figure 2B). Samples from CHO137 lysates were also run from an independent time course and separated for 4E-BP1 western analysis at greater resolution in order to visualise the different bands more clearly.

### Phosphorylation of other regulators of translation during batch culture

**Figure 3.**
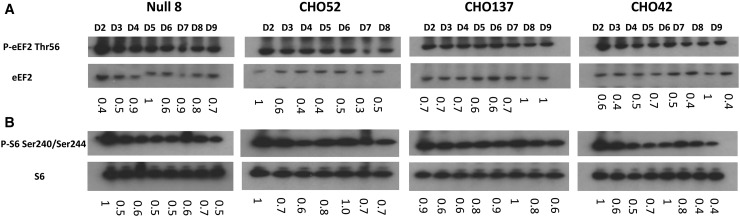
Direct and indirect mTOR-mediated translational control mechanisms. (**A**) P**-**eEF2 Thr56 phospho-isoform and eEF2 examined by western blot analysis on stated days (D). (**B**) P**-**S6 Ser240/Ser244 phospho-isoform and S6 total examined by western blot analysis on stated days (D). For both set of gels, the ratio between eEF2-P/eEF2 and S6-P/S6 was calculated. The highest ratio value was set to 1 and used as the reference.

mTORC1-promoted phosphorylation of S6Ks may enhance translation via several possible mechanisms, including via inactivation of the kinase that phosphorylates eukaryotic elongation factor eEF2 [25,26]. We therefore assessed the phosphorylation profile of eEF2 and S6 (a direct substrate for S6Ks) during batch culture in the different cell lines (Figure 3). Phosphorylation of elongation factor eEF2 at threonine 56 inhibits its activity. Although S6Ks phosphorylate ribosomal protein S6, it is not clear whether or how this affects mRNA translation. S6Ks also phosphorylate eIF4B, an event which is thought to increase its assembly in the translation initiation complex and thus stimulate translation initiation [26]. S6Ks (S6K1 and 2) have also been linked to promoting ribosome biogenesis [27]. eEF2 phosphorylation levels remained more or less unchanged in the cell lines investigated (see Figure 3A), with the exception of CHO52 where a change is observed at the beginning of the time course (this may reflect the fact that these cells are more susceptible to stress when subjected to sub-culturing). Towards the end of the batch culture, a greater proportion of total eEF2 was generally phosphorylated in the CHO cells as indicated by the ratio of phosphorylated to total eEF2 (Figure 3A). On the other hand, S6 phosphorylation tended to peak approximately mid-culture and declined towards the end of the batch culture (Figure 3B).

### The ratio of 4E-BP1 and eIF4E correlates with recombinant antibody yields as determined in GS-CHOK1SV cell lines

**Figure 4.**
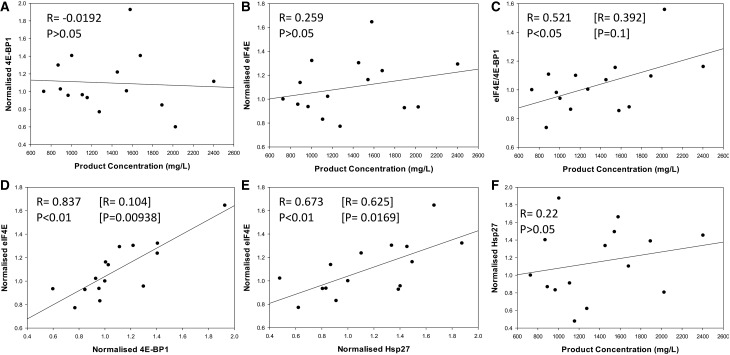
**Relationship of 4E-BP1, eIF4E, 4E-BP1:eIF4E, and Hsp27 to product concentration and each other in recombinant antibody producing Chinese hamster ovary cell lines.** (**A**–**F**) The relative levels of 4E-BP1, eIF4E and Hsp27 were measured in 15 different cell lines that exhibit differential recombinant protein productivity. The correlation coefficients between 4E-BP1 or Hsp27 levels and recombinant protein product concentration and *P*-values were calculated using SigmaPlot. Values in brackets correspond to recalculated *R* and *P*-values in the absence of the outlier(s) identified by Cook's distance. Outliers were defined as such if they were above three times the average Cook's distance.

Following our observation that 4E-BP1 and eIF4E were elevated in the CHO52 cell line compared with the Null8, CHO137 and CHO42 cell lines, we first sought to determine whether there was an inverse relationship between low (or high) productivity and the amounts of 4E-BP1 and eIF4E. We then asked whether the ratio of eIF4E:4E-BP1 rather than individual protein levels correlated with product yields from GS-CHOK1SV cells. Previous studies by Alain et al. [28,29] established that the stoichiometry between eIF4E and 4E-BP1 influences neoplastic growth. To determine how the variables (amounts of eIF4E and 4E-BP1 versus cell productivity, eIF4E levels versus 4E-BP1 levels) related to each other, we generated scatterplots and undertook correlation analysis using a Pearson's correlation coefficient approach (Figure 4A–F). For this analysis, we investigated 15 different GS-CHOK1 mAb-producing sister cell lines generated from the same transfection and the cell line construction process [22,23]. The resulting analysis (Figure 4C) showed that the eIF4E/4E-BP1 ratio was correlated to cell productivity (correlation coefficient *R* = 0.521; *P* < 0.05) when all cell lines were considered. When we performed a Cook's distance test to identify any data points that were outliers or that might distort the regression analysis, one cell line was identified as an outlier and when ignored in the analysis, the confidence was reduced at 90%. Interestingly, the amounts of 4E-BP1 correlated well with those of eIF4E (*R* = 0.837, *P* < 0.01, Figure 4D). When we removed the outlier, identified by Cook's distance analysis, the *P*-value was <0.001 (Figure 4D). This suggests the possibility that there is a co-regulation between eIF4E and 4E-BP1 amounts.

### Amounts of the chaperone Hsp27 correlate with eIF4E levels

Folding of newly synthesised polypeptides into the correct, active 3D shape is often assisted by proteins commonly termed chaperones, which include the heat shock proteins (Hsps). In addition, small Hsps can exert a protective role on their substrate. Of particular interest here, Andrieu et al. [30] showed that Hsp27 can interact with eIF4E and protect it from degradation. We therefore investigated whether there was a correlation between eIF4E amounts and Hsp27 amounts in our 15 model CHO cell lines. Our analysis in Figure 4E confirmed that not only did the amounts of eIF4E correlate with those of 4E-BP1 (Figure 4D), but also with Hsp27 levels (Figure 4E, *R* = 0.673, *P* < 0.01). The correlation coefficient (*R* = 0.625) and *P*-value were slightly lower (*P* = 0.0169) when we removed the outlier identified by Cook's distance (Figure 4E).

We have previously conducted a study on chaperone-assisted rPP in CHO cell lines [10]. This work revealed that transient overexpression of Hps27 could lead to enhanced yields of recombinant cytoplasmic Firefly luciferase but not a recombinant secreted *Gaussia* luciferase in CHOK1 cells. We therefore investigated the expression profile of Hsp27 (Supplementary Figure S1) across batch culture in the Null8, low- and high-producer cell lines from our original panel. In the Null8 cell line, Hsp27 remained more or less the same across the time course. However, Hsp27 levels clearly decreased as batch culture proceeded in the low (CHO52) and high (CHO137 and CHO42) producers (Supplementary Figure S1).

### Expression of 4E-BP1 and eIF4E are co-regulated in GS-CHOK1SV cells, but changes in the eIF4E/4EBP1 ratio do not significantly influence recombinant mAb production

**Figure 5.**
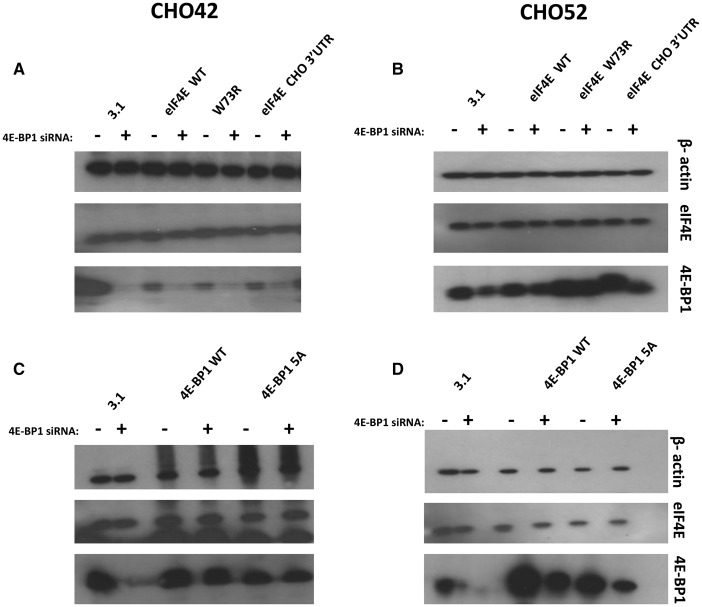
**Modulation of eIF4E/4E-BP1 ratios in low (CHO42) and high (CHO52) producers**. (**A–D**) CHO cells were transfected with human eEF4E variants together with mock siRNA (−) or siRNA (+) directed at 4E-BP1. Samples of cell lysate were analysed by western blot for the indicated proteins.

We then further investigated the potential co-regulation of 4E-BP1 and eIF4E and whether a ‘low-producing cell line’ could be engineered towards a ‘good producing cell line’ (and vice versa) if the endogenous stoichiometry between these two factors was modified. To manipulate 4E-BP1 levels, 4E-BP1 was either knocked down via siRNA-mediated silencing or transiently overexpressed (Figure 5A–D). To allow for detection of exogenous protein levels, the 4E-BP1 cognate clones bore a c-Myc tag. We tested two variants, wild-type 4E-BP1 and a non-phosphorylatable dominant-negative mutant, where the serine/threonine sites are mutated to alanine [30,31]. Our results show that reducing 4E-BP1 levels was accompanied by a decrease in the total levels of eIF4E in both the high- (CHO42) and low- (CHO52) producing cell lines (Figure 5A,B). These data support the co-regulation of amounts of eIF4E with 4E-BP1 levels in CHO cells. Notably, the levels of total 4E-BP1 were lower when the dominant-negative variant was introduced in both CHO42 and CHO52 compared with wild-type, although this effect was more marked in the latter (Figure 5D,E). This result suggests that the non-phosphorylated form is more prone to degradation than the wild type, at least when present in high amounts in the cell. A study by Yanagiya et al. [32] proposed that the hypophosphorylated form of 4E-BP1 is less stable than the hyperphosphorylated form when eiF4E was silenced. However, the levels of eIF4E remained unchanged in this experiment.

We also generated a series of human eIF4E (heIF4E) variants to assess the effect of heIF4E overexpression on 4E-BP1 levels. The constructs were V5-tagged to discriminate between endogenous and exogenous eIF4E. Because overexpression of any protein potentially has an impact on the translation machinery, we also created two mutants of heIF4E, [W73R] and [V69G], which have previously been reported [33]. heIF4E [W73R] and V69G cannot bind to eIF4G (or to 4E-BP1) and therefore cannot initiate translation at the mRNA cap. As the amounts of eIF4E are known to be regulated by many factors (e.g. AU-rich elements in the eIF4E 3′-UTR mediate binding of HuR to the eIF4E mRNA and its stabilisation [34]), we modified the heIF4E wild-type constructs by adding the hamster eIF4E 3′-UTR. We found that there was no detectable difference in eIF4E amounts when exogenous eIF4E-encoding plasmids were introduced into CHO cells when probing for total eIF4E (Supplementary Figure S2A,B). Detection of exogenously expressed eIF4E variants via the V5-tag showed that these variants were expressed (wild-type and 3′-UTR variants) or not detected at all (W73R variant) in CHO cells (Supplementary Figure S2A,B). Due to use of the V5-tag antibody to detect the exogenous variants, it is not possible to determine how the amounts of exogenous material directly compare with the endogenous material. In Figure 5C,D, overexpression of 4E-BP1 did not result in enhanced eIF4E protein amounts, suggesting again that the amount of eIF4E is tightly regulated or that the amount of exogenous 4E-BP1 expressed was insignificant/not sufficient to influence eIF4E amounts. One of the aims of this analysis was to assess whether increase in the eIF4E/4E-BP1 ratio could improve the productivity of low-producing cell lines such as CHO52; however, this proved difficult to achieve in this cell line at least and we could not detect any change in the overall rate of translation between the conditions tested as inferred from ^35^S labelling of new proteins (Supplementary Figure S3A,B). We also performed an ELISA assay to compare the production of IgGs from CHO42 and CHO52 in the presence of either 4E-BP1 siRNA or V5-tagged IF4E (data not shown). No significant difference in antibody was detected at 48 or 72 h post-transfection, demonstrating the complexity of regulation of 4E, 4E-BP1 and rPP, and that overexpressing or down-regulating individual members of a highly regulated network does not necessarily result in a modification of the desired phenotype.

### 4E-BP1 levels are influenced by PPM1G amounts

**Figure 6.**
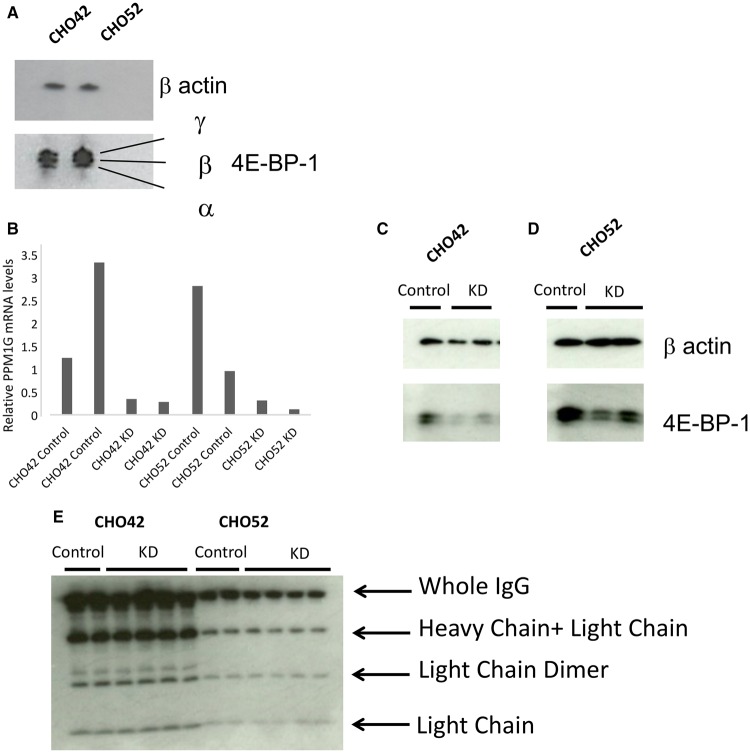
Role of PPM1G in modulating 4E-BP1 activity. (**A**) 4E-BP1 isoforms in CHO. (**B**) PPM1G knockdown (KD), mRNA levels were normalised to the β-actin mRNA. (**C** and **D**) Protein levels in control or silenced (KD) samples. (**E**) Effect of PPM1G knockdown on recombinant protein expression (IgG/mAb expression).

4E-BP1 exhibits different phosphorylation states and the phosphorylation varies depending on environmental conditions (see Figure 2C). However, under standard physiological conditions, the low-producer (CHO52) and high-producer (CHO42) cell lines had a similar 4E-BP1 phosphorylation profile (Figure 6A), although the ratio of eIF4E:4E-BP1 was different. Hypophosphorylated forms of 4E-BP1 have been shown to exhibit a faster degradation rate than higher molecular mass hyperphosphorylated isoforms [35]. Previously, Liu et al. [36] showed that PPM1G, a member of the protein phosphatase 2C family, can influence the phosphorylation state of 4E-BP1 independently of mTORC1. Silencing of PPM1G is reported to result in increased luciferase expression in HEK293E cells, presumably reflecting higher amounts of phosphorylated, inactive 4E-BP1. We therefore investigated whether changing PPM1G amounts influenced 4E-BP-1 phosphorylation and amounts, and influenced rPP, in the low- (CHO52) and high- (CHO42) producing cell lines. mRNA levels were measured by qPCR and normalised to three housekeeping calibrators (β-actin, β2-microglobulin and GAPDH). In the absence of knockdown (control samples), PPM1G amounts varied between replicates, presumably reflecting biological variation between samples; however, in knockdown samples in the presence of siRNA, the observed PPM1G mRNA amounts were consistent and reduced compared with the controls (Figure 6B). When we reduced the amount of PPM1G by siRNA (Figure 6B–D), the phosphorylation pattern of 4E-BP1 was only marginally changed compared with the control, but the actual levels of 4E-BP1 were greatly reduced. This suggests that amounts of PPM1G are also important in modulating 4E-BP1 levels in CHO cells, which in turn are co-regulated with eIF4E amounts. However, this reduction in total 4E-BP1 had no positive effect on the amount of mAb protein being secreted from the producing cell lines (Figure 6E).

### Does eIF4E and 4E-BP1 subcellular localisation relate to productivity of CHO cells?

Lejbkowicz et al. [37] showed that while eIF4E is predominantly cytoplasmic, in mammalian cells a significant fraction (12–33%) localises to the nucleus, where it appears to co-localize with splicing factors [38]. The nuclear import of eIF4E is mediated by 4E-T (eIF4E transporter), which binds to eIF4E and simultaneously interacts with the nuclear import receptors importin α/β [39]. It is thought that nuclear eIF4E helps promote the export from the nucleus of a subset of mRNAs [40]. Furthermore, Rong et al. [41] showed that around a third of endogenous 4E-BP1 is localised to the nucleus in mouse embryo fibroblasts and that 4E-BP1 can regulate the subcellular localisation of eIF4E. Since our work showed (i) that the total levels of 4E-BP1 correlated with those of eIF4E, (ii) that the eIF4E/4E-BP1 ratio weakly correlates with cell productivity and (iii) that knockdown of 4E-BP1 resulted in a reduction in eIF4E amounts, we examined whether the distribution of eIF4E and 4E-BP1 was different in the low- (CHO52) and high-producer (CHO42) cell lines. Immunofluorescence, using antibodies against 4E-BP1 or eIF4E (Supplementary Figure S4), showed little difference in the localisation or intensity of the signal for the two proteins between the cell lines.

## Discussion

Here, we have investigated selected effectors of the mTORC1 signalling pathway that are involved in the control of protein synthesis in commercially relevant GS-CHOK1SV mAb-producing cell lines. In particular, we have investigated the relationship between 4E-BP1 amounts and phosphorylation and eIF4E amounts. Although the signalling pathways that converge on the mTORC1 kinase as well as mTORC1 downstream effectors have been widely reported, few studies have focused on mTORC1 regulation in rPP systems. Recent findings have implied that the mTORC1 signalling network could be exploited in bioprocessing. For example, exogenous expression of the mTOR kinase in CHO cell lines led to increased recombinant IgGs yields [20], although this study did not measure the amount of, or confirm, exogenous expression. Dadehbeigi and Dickson [22] also showed that inhibition of growth and rP titre in rapamycin-treated cells was transient, while 4E-BP1 phosphorylation remained stable. These two sets of results highlight that the interpretation of mTORC1 regulation in the context of rPP is complex [22]. More recently, Edros et al. [42] performed a transcriptomic analysis on a large panel of factors related to mTORC1 signalling in two different recombinant CHO cell lines with a 17.4-fold difference in mAb productivity. They showed that, across this pool of 84 genes, eight genes exhibited differences of >1.5-fold. These included upstream regulators of mTORC1 (AMPK, phospholipase D and Ras-related GTP-binding protein C) and one mTOR effector (the ribosomal protein S6). However, the present study only highlighted transcripts (mRNA levels), whereas other post-transcriptional and post-translational control mechanisms govern the mTOR network.

Besides cell proliferation, mTORC1 regulates diverse pathways including autophagy, a process which recycles broken-down intracellular components. The proteins ULK1, Atg13 and FIP200 complex link mTORC1 signalling to autophagy [43]. Our study indicates that a low-producing cell line (CHO52), where cell viability decreases during batch culture earlier than other cell lines, exhibits markers of autophagy (conversion of LC3-I into LC3-II) at an earlier stage compared with those cell lines where viability does not decrease at similar times (including both higher producing cell lines and a Null-producing cell line). Others have reported that autophagy can be beneficial for cell survival [24]. These data collectively suggest that a balance between maintaining cell viability and the benefits of ‘recycling’ of cellular components late in culture together can influence recombinant protein output. However, our data also suggest that activation of autophagy in a low-producing cell line is not helpful in terms of productivity as it is associated with a lower protein synthesis rate and a decline in viable cell numbers in the cell lines investigated here.

Previous reports have suggested that the yield of recombinant proteins from cultured mammalian cells is in part attributed to translation efficiency [8,44]. mTORC1 exerts its influence on translation via the mTORC1 effectors 4E-BP1, p70 S6 kinase and eEF2K. While we observed that 4E-BP1 amounts could differ across recombinant protein-producing cell lines, 4E-BP1 amounts alone did not correlate to cell productivity. Rather, the protein ratio of 4E-BP1 to eIF4E, a central parameter in cap-dependent translation, showed a higher degree of correlation with cell productivity. Furthermore, eIF4E amounts appear to be co-regulated with changing 4E-BP1 amounts when 4E-BP1 was reduced by knockdown experiments, but not when 4E-BP1 was reduced by manipulation of the PPM1G phosphatase. This may reflect a sensing of either change at the mRNA level as opposed to the protein level or the fact that the phosphorylation status and the amount of 4E-BP1 present are important in the co-regulation of eIF4E with 4E-BP1. With respect to 4E-BP1 phosphorylation, mammalian cells contain multiple 4E-BP1 isoforms, but the isoform pattern observed in rodent cells is simpler [43,44]. Consistent with the rat model, our work and that of others [45] show that in CHO cells, a total of three 4E-BP1 species are usually detected: a hyperphosphorylated form γ, a middle form β and a hypophosphorylated α form (see Figures 2B and 6A). However, lower resolution acrylamide/bis-acrylamide gels may only reveal two bands. The intensity of each band probably reflects the physiological state of the cell. Indeed, as inferred by the m^7^-agarose-binding experiment (Figure 2A), the phosphorylation status of 4E-BP1 is altered through the batch culture time course. mTOR-mediated phosphorylation of 4E-BP1 is a precisely orchestrated process, with phosphorylation in human 4E-BP1 on Thr37 and Thr46, seemingly acting as a priming event for subsequent phosphorylation on other serine/threonine sites [30, 31]. These events are generally thought to influence the affinity of 4E-BP1 for eIF4E and, in this way, regulate translation initiation, although this model has recently been disputed by Showkat et al. [46].

As 4E-BP1's state of phosphorylation has an impact on translation initiation, we wished to explore whether changing the amounts of 4E-BP1 as a whole or the isoforms with respect to each other would lead to discrete changes in recombinant protein synthesis. mTORC1 catalyses the phosphorylation of 4E-BP1, but also regulates many other cellular events; therefore, manipulating mTOR-mediated phosphorylation may lead to diverse consequences. Liu et al. [36] performed lentivirus-mediated silencing of a series of serine/threonine phosphatases and identified PPM1G as a phosphatase for 4E-BP1. We therefore induced PPM1G knockdown via siRNA-mediated silencing (Figure 6B) and observed a large drop in the overall amount of 4E-BP1, but only a subtle change in the phosphorylation pattern (the highest phosphorylated form being more abundant; Figure 6C,D). This may indicate that artificially altering the ‘standard’ mTOR-driven sequence of phosphorylation events in 4E-BP1 can lead to 4E-BP1 instability. Notably, the levels of eIF4E were unchanged (data not shown).

Analysis of IgG molecules by western blot (Figure 6E) showed that there was no increase in product yield following PPM1G knockdown, which is in contrast with the observations of Liu et al. [36]. Even if PPM1G knockdown led to an overall decrease in 4E-BP1, this should not significantly affect the amount of IgG being secreted from the cells, as we found that 4E-BP1 amounts did not correlate with cell productivity (Figure 4). Finally, as inferred by our [^35^S]methionine labelling experiment (Supplementary Figure S3), 4E-BP1 knockdown or overexpression at the levels achieved in the present study are not sufficient to reprogram the core capacity of a cell to undertake translation, this being observed in both high- (CHO42) or low- (CHO52) producing cell lines. This finding demonstrates that manipulation of single components within a complex regulatory process does not necessarily lead to a change in phenotype.

Another noteworthy finding in our study was that the levels of the chaperone Hsp27 declined over the time course. This was not observed in the non-producing cell line Null8. Tan et al. [47] observed that stably expressing Hsp27 could improve rPs yield in cells. Interestingly, when we looked at our panel of 15 recombinant cell lines, we saw no correlation between Hsp27 and cell productivity (Figure 4). However, Hsp27 levels correlated with eIF4E levels (Figure 4). A study by Cuesta et al. [48] suggested that Hsp27 targets eIF4E and eIF4G and attenuates protein translation in stressed cells by preventing the assembly of the cap initiation complex. Andrieu et al. [30] demonstrated that Hsp27 directly interacted with eIF4E. They suggested that Hsp27 has a protective role over eIF4E, thus protecting protein synthesis initiation. The exact mode of action of Hsp27 at the cap-binding stage remains unclear.

Our study highlights that the stoichiometry between the mTORC1 effector 4E-BP1 and the translation factor eIF4E differs across CHO recombinant cell lines and correlates with cell productivity. Interestingly, Yanagiya et al. [32] showed that a 90% reduction in eiF4E at the mRNA level only resulted in partial inhibition of mRNA translation. The same study shows that the 4E-BP1 hypophosphorylated form was less prone to degradation than hyperphosphorylated 4E-BP1, when eiF4E was knocked down. Our work shows that the stoichiometric balance between eiF4E and 4E-BP1 is constrained by the expression for each factor, stability, phosphorylation status, physical interaction and cellular localisation, which means that simply correcting the level of 4E-BP1 or eIF4E in the cells is not sufficient to convert a low-producing cell line into a high-producing cell line. Our analysis suggests that additional factors, such as eIF4G isoforms and Hsp27, are also likely to play a role in the tuning of this balance, and hence, upstream regulators of overall mTORC1 signalling are more likely to be targets whose manipulation can be utilised to drive cell phenotypes for enhanced rPP.

## Abbreviations

4E-BP1: 4E-binding protein 1
CHO: Chinese hamster ovary
heIF4E: human eIF4E
Hsps: heat shock proteins
HuR: human antigen R protein or ELAV-like protein 1
IVC: integral of viable cell concentration
m^7^GTP: γ-aminophenyl-7-methyl-guanosine 5′-triphosphate
mAb: monoclonal antibody
mTOR: mammalian target of rapamycin
mTORC1: mTOR complex 1
rPP: recombinant protein production
rPs: recombinant proteins
S6K1: S6 protein kinase 1.
